# Association between antidepressant use and ED or hospital visits in outpatients with SARS-CoV-2

**DOI:** 10.1038/s41398-022-02109-3

**Published:** 2022-08-22

**Authors:** Bradley A. Fritz, Nicolas Hoertel, Eric J. Lenze, Farid Jalali, Angela M. Reiersen

**Affiliations:** 1 Department of Anesthesiology, Washington University School of Medicine, St. Louis, MO USA; 2 Assistance Publique-Hôpitaux de Paris, Hôpital Corentin-Celton, 92130 Issy-les-Moulineaux, France; 3 Université Paris Cité, Paris, France; 4 Institut de psychiatrie et neurosciences de Paris (IPNP), INSERM, UMR_S1266, Paris, France; 5 Department of Psychiatry, Washington University School of Medicine, St. Louis, MO USA; 6 Department of Gastroenterology, Saddleback Medical Group, Laguna Hills, CA USA

**Keywords:** Depression, Biomarkers

## Abstract

Antidepressants have previously been associated with better outcomes in patients hospitalized with COVID-19, but their effect on clinical deterioration among ambulatory patients has not been fully explored. The objective of this study was to assess whether antidepressant exposure was associated with reduced emergency department (ED) or hospital visits among ambulatory patients with SARS-CoV-2 infection. This retrospective cohort study included adult patients (*N* = 25 034) with a positive SARS-CoV-2 test performed in a non-hospital setting. Logistic regression analyses tested associations between home use of antidepressant medications and a composite outcome of ED visitation or hospital admission within 30 days. Secondary exposures included individual antidepressants and antidepressants with functional inhibition of acid sphingomyelinase (FIASMA) activity. Patients with antidepressant exposure were less likely to experience the primary composite outcome compared to patients without antidepressant exposure (adjusted odds ratio [aOR] 0.89, 95% CI 0.79–0.99, *p* = 0.04). This association was only observed with daily doses of at least 20 mg fluoxetine-equivalent (aOR 0.87, 95% CI 0.77–0.99, *p* = 0.04), but not with daily doses lower than 20 mg fluoxetine-equivalent (aOR 0.94, 95% CI 0.80–1.11, *p* = 0.48). In exploratory secondary analyses, the outcome incidence was also reduced with exposure to selective serotonin reuptake inhibitors (aOR 0.87, 95% CI 0.75–0.99, *p* = 0.04), bupropion (aOR 0.70, 95% CI 0.55–0.90, *p* = 0.005), and FIASMA antidepressant drugs (aOR 0.87, 95% CI 0.77–0.99, *p* = 0.03). Antidepressant exposure was associated with a reduced incidence of emergency department visitation or hospital admission among SARS-CoV-2 positive patients, in a dose-dependent manner. These data support the FIASMA model of antidepressants’ effects against COVID-19.

## Introduction

The novel coronavirus SARS-CoV-2 and its variants have created a worldwide infectious disease crisis in the form of the coronavirus disease 2019 (COVID-19) pandemic [1]. Because many individuals around the world have not been fully vaccinated [2] and breakthrough infections can sometimes cause severe COVID-19 in vaccinated individuals [3], effective treatments with favorable tolerability profiles are urgently needed. Ideal treatments would be easy to use, low in cost, produced in oral formulations, and rapidly available globally, including in low and middle income countries. Repurposing of existing drugs may be imperative to identifying such therapeutics [4, 5].

Certain antidepressants, particularly selective serotonin reuptake inhibitors (SSRIs), have shown promise as early treatments for COVID-19 [6, 7]. Multiple preclinical studies have demonstrated in vitro efficacy of both SSRIs and non-SSRI antidepressants against SARS-CoV-2 in human and non-human host cells [8–12]. Potential mechanisms of action include immunomodulatory activity via sigma-1 receptor (S1R) agonism and non-S1R pathways (e.g., NF-κB, inflammasomes, TLR4, PPARγ) [13–15], antiviral and anti-inflammatory actions via functional inhibition of acid sphingomyelinase (FIASMA) activity [10, 16–18], as well as serotonin modulatory and anti-platelet activity of these agents. Among patients with COVID-19 treated in acute care settings, three large retrospective observational cohort studies have reported reduced death or mechanical ventilation among patients with antidepressant exposure [19–21]. In these studies, the association with improved outcomes appeared to be strongest among patients taking the SSRIs fluoxetine or fluvoxamine [19–21]. In the ambulatory setting, the use of fluvoxamine for 10-15 days was associated with a reduced risk of clinical deterioration in two randomized, placebo-controlled trials [22, 23] as well as in one non-randomized observational study [24]. However, the impact of antidepressants other than fluvoxamine have not been investigated in the ambulatory setting. In addition, prior studies did not examine a potential dose-response relationship between antidepressant use and reduced risk of developing severe COVID-19. Therefore, the goal of this study was to test the hypothesis that exposure to antidepressants would be associated with reduced incidence of clinical deterioration, defined as emergency department (ED) visitation or hospital admission, among ambulatory patients with COVID-19.

## Methods

This retrospective cohort study was approved by the Human Research Protection Office at Washington University School of Medicine. A waiver of informed consent was obtained to retrieve data from the electronic health record. This study was conducted at BJC Healthcare, a network of university-affiliated hospitals and community hospitals located in Missouri and Illinois. This manuscript has been written according to STROBE guidelines [25].

The population included patients age 18 or older with a positive SARS-CoV-2 test (either polymerase chain reaction or antigen test) performed within the BJC Healthcare network between 3/1/2020 and 5/16/2021. Because the outcome was ED visitation or hospital admission, patients who were admitted to a hospital at the time of the test or admitted on the same day as the test were excluded from the analysis.

Outpatient medication exposure was determined by extracting the home medication list as documented in the electronic health record of all ambulatory or inpatient encounters within the study period. For each medication of interest, exposure was defined as the inclusion of the medication on the home medication list at any encounter prior to the date of the first positive SARS-CoV-2 test. The full list of medications retrieved is found in Table S1. For antidepressants, the strength of the oral formulation was also recorded. The prescription sig (patient instruction) data were unavailable, but daily doses were approximated from the formulation strength by assuming that one tablet/capsule was taken once daily.

To explore potential mechanisms of action, antidepressants were first stratified by class (i.e., SSRIs, serotonin-norepinephrine reuptake inhibitors, tricyclic antidepressants, phenylpiperazines). Then, SSRIs were grouped based on their activity at the S1R [26]: high-affinity agonists (fluoxetine, fluvoxamine), intermediate-affinity agonists (escitalopram, citalopram), low-affinity agonist (paroxetine), and antagonist (sertraline). Finally, antidepressants were classified as drugs with FIASMA activity, defined as showing an in vitro functional inhibition effect on acid sphingomyelinase (ASM--i.e., a residual ASM activity <100%) [10, 27], including amitriptyline, citalopram, clomipramine, desipramine, doxepin, escitalopram, fluoxetine, fluvoxamine, imipramine, nortriptyline, paroxetine, sertraline, and venlafaxine, and antidepressants with unknown FIASMA, defined as unknown residual ASM activity after prolonged (e.g., >6 h) incubation times, including bupropion, desvenlafaxine, duloxetine, levomilnacipran, mirtazapine, nefazodone, trazodone, vilazodone, and vortioxetine.

The primary outcome was a composite of emergency department (ED) visitation or hospital admission within 30 days after the patient’s first positive SARS-CoV-2 test. Encounters at any ED or hospital in the BJC network were identified.

### Statistical methods

All analyses were conducted using R version 4.0.3 (R, Vienna, Austria). Code is available at https://github.com/bafritz/covidantidepressant. *P* values < 0.05 were considered to be statistically significant. *P* values were not adjusted for multiple comparisons, and secondary analyses should be interpreted as exploratory. No sample size calculation was performed, as all patients with available data were included. The cohort was divided into two groups: patients with outpatient exposure to an antidepressant and those with no outpatient exposure to an antidepressant. Demographic characteristics, elements of the medical history, and home medication characteristics were compared between these two groups using chi-square tests or Wilcoxon rank-sum tests. The dose range for each individual antidepressant was described using median, interquartile range, and range. To permit comparisons of dose ranges across medications with different potencies, strengths were converted to fluoxetine-equivalents using the conversion factors based on prior work [28] (Table S2).

Crude and adjusted odds ratios for ED or hospital encounters within 30 days were estimated using logistic regression. The primary analysis compared patients exposed to any antidepressant against patients exposed to no antidepressant. Medical comorbidity and concurrent use of other psychotropic medications are more prevalent among patients hospitalized for COVID-19 with psychiatric disorders [19, 29–31], and these patients are more likely both to take antidepressants and to develop severe COVID-19 [32, 33]. Therefore, the logistic regression adjusted for sex, age, race and ethnicity, obesity, Elixhauser comorbidity index [34], history of mood or anxiety disorder, history of other psychiatric disorders, number of home medications, and concurrent exposure to benzodiazepines or Z drugs (eszopiclone, zaleplon, or zolpidem), to antipsychotic drugs, and to non-antidepressant drugs with FIASMA [35] or S1R activity [36]. Model discrimination was quantified using the C statistic, and calibration was assessed using a calibration plot. For each variable in the model, the variance inflation factor was used to measure multicollinearity with other variables in the model. A sensitivity analysis was conducted excluding influential outliers, defined as observations with standardized residuals >3 and Cook’s distance > 4/*n* (where *n* is the number of observations in the model). In secondary analyses, crude and adjusted odds ratios for each individual antidepressant and for each class of antidepressants were estimated using the above-mentioned methods. As an additional secondary analysis, we examined a potential dose-effect relationship by grouping patients according to their daily antidepressant dose in fluoxetine-equivalents (<20 mg, ≥20 mg, and ≥40 mg). Crude and adjusted odds ratios for the primary outcome were also calculated for each of these groups. In each secondary analysis, the exposed patients were compared to patients with no antidepressant exposure (excluding patients exposed to different antidepressants).

## Results

Between 3/1/2020 and 5/16/2021, 25 034 outpatients were found to have a positive SARS-CoV-2 test result. Of these patients, 4557 cases (18.3%) had exposure to at least one antidepressant documented in the home medication list, at a median fluoxetine-equivalent daily dose of 22.2 mg (interquartile range 20.0–42.0 mg). Patients with antidepressant exposure were older, more likely to be female, and more likely to be of White race and non-Hispanic ethnicity compared to patients without antidepressant exposure (Table 1). Additionally, the patients with antidepressant exposure were more likely to take a greater number of home medications, including any benzodiazepine or Z-drug and any antipsychotic, and to have diagnoses of mood or anxiety disorder or other psychiatric disorders. The most common classes of antidepressants included SSRIs (2744 patients, 60.0%), serotonin-norepinephrine reuptake inhibitors (994 patients, 21.7%), and phenylpiperazines (761 patients, 16.6%) (Table 2).

**Table 1:** Characteristics of patients with and without antidepressant exposure.

Variable	Number with missing data	Overall (*N* = 25,034)		With antidepressant exposure (*N* = 4577)		Without antidepressant exposure (*N* = 20,457)		*P*^a^
		N or Median	% or IQR	N or Median	% or IQR	N or Median	% or IQR	
Age (median, IQR)	0	47	32–61	53	39–65	46	31–60	<0.001
Sex	18							<0.001
Female		15,030	60.1	3383	73.9	11,647	57	
Male		9986	39.9	1194	26.1	8792	43	
Race	1250							<0.001
American Indian/Alaska Native		51	0.2	10	0.2	41	0.2	
Asian		275	1.2	27	0.6	248	1.3	
Black or African American		6389	26.9	716	15.8	5673	29.5	
Other		7	<0.1	0	0	7	0	
Other Pacific Islander		54	0.2	9	0.2	45	0.2	
White		17,008	71.5	3782	83.2	13,226	68.7	
Ethnicity	2909							<0.001
Hispanic		440	2.0	55	1.2	385	2.2	
Non-hispanic		21,685	98.0	4376	98.8	17,309	97.8	
Obese	3234	11,197	51.4	2619	57.5	8578	49.7	<0.001
Elixhauser Index (median, IQR)	1854	0	0–0	0	−4 to 3	0	0–0	<0.001
Mood or anxiety disorder	1854	5884	25.4	3283	71.7	2601	14	<0.001
Other psychiatric disorder	1854	2417	10.4	1057	23.1	1360	7.3	<0.001
Number of home medications	0							<0.001
0		11,149	44.5	0	0	11,149	54.5	
01–05		4736	18.9	788	17.2	3948	19.3	
06–10		3435	13.7	1036	22.6	2399	11.7	
11–15		2382	9.5	928	20.3	1454	7.1	
16+		3332	13.3	1825	39.9	1507	7.4	
Benzodiazepine or Z drug	0	1997	8	1181	25.8	816	4	<0.001
Antipsychotic drug	0	582	2.3	452	9.9	130	0.6	<0.001
Non-antidepressant drug with FIASMA activity	0	4982	19.9	1949	42.6	3033	14.8	<0.001
Non-antidepressant drug with S1R activity	0	153	0.6	101	2.2	52	0.3	<0.001

*FIASMA* functional inhibition of acid sphingomyelinase, *IQR* interquartile range, *S1R* sigma-1 receptor.

^a^*P* values calculated using Wilcoxon rank-sum test for age and Elixhauser Index. *P* values calculated using chi square test for all other variables.

**Table 2:** Antidepressants used and range of formulation strengths.

Antidepressant	Number of patients	Strength (mg)			Strength (mg Fluoxetine-equivalent)		
		Median	IQR	Min to max	Median	IQR	Min to max
Any Antidepressant^a^	4577				22.2	20–42	2.9–158
Any SSRI	2744				22.2	21–42	8.4–120
Fluoxetine	403	20	20–40	10–90	20	20–40	10–90
Fluvoxamine	17	50	50–100	25–100	15	15–30	7.5–30
Citalopram	507	20	20–40	10–40	22.2	22.2–44.4	11.1–44.4
Escitalopram	812	10	10–20	5–20	22.2	22.2–44.4	11.1–44.4
Paroxetine	179	20	10–30	10–40	23.4	11.7–35.1	11.7–46.8
Sertraline	843	50	50–100	20–100	21	21–42	8.4–42
Vilazodone	38	20	20–40	10–40	30	30–60	15–60
Vortioxetine	52	10	10–20	5–20	30	30–60	15–60
Any Non-SSRI	2591				16.5	8.25–33	0.87–108.8
Any TCA	567				8.25	4–16.5	0.87–63.5
Amitriptyline	333	25	10–50	10–150	8.25	3.3–16.5	3.3–49.5
Clomipramine	7	50	50–62.5	25–75	17.5	17.5–21.9	8.75–26.25
Desipramine	8	37.5	25–50	10–50	7.875	5.25–10.5	2.1–10.5
Doxepin	31	25	10–25	3–150	7.25	2.9–7.25	0.87–43.5
Imipramine	12	25	17.5–50	10–75	7.25	5.07–14.5	2.9–21.75
Nortriptyline	188	25	10–25	10–75	10	4–10	4–30
Any Phenylpiperazine	761				5	5–10	5–30
Nefazodone	2	100	100–100	100–100	8	8–8	8–8
Trazodone	760	50	50–100	50–300	5	5–10	5–30
Any SNRI	994				40	20.1–40.2	7–103.2
Desvenlafaxine	46	50	50–100	25–100	20	20–40	10–40
Duloxetine	559	60	30–60	20–60	40.2	20.1–40.2	13.4–40.2
Levomilnacipran	4	100	60–120	40–120	33	19.8–39.6	13.2–39.6
Venlafaxine	400	75	75–150	25–225	21	21–42	7–63
Other Antidepressant	750						
Bupropion	589	150	150–300	75–450	16.5	16.5–33	8.25–49.5
Mirtazapine	165	15	7.5–30	7.5–45	11.85	5.9–23.7	5.9–35.6
SSRIs GROUPED BY S1R AFFINITY^b^							
High affinity	418				20	20–40	10–90
Intermediate affinity	1307				22.2	22.2–44.4	11.1–88.8
Low affinity	179				23.4	11.7–35.1	11.7–46.8
ANTIDEPRESSANTS GROUPED BY FIASMA ACTIVITY							
With FIASMA activity^c^	3414				22.2	20–42	2.1–101.4
With unknown FIASMA activity^d^	1904				16.5	10–35.3	5–158

*FIASMA* functional inhibition of acid sphingomyelinase, *IQR* interquartile range, *SNRI* serotonin-norepinephrine reuptake inhibitor, SSRI selective serotonin reuptake inhibitor, *S1R* sigma-1 receptor, TCA tricyclic antidepressant.

^a^For patients taking more than one antidepressant, the strength of each medication was converted to fluoxetine-equivalents, and then these converted strengths were added together.

^b^SSRIs with high-affinity agonist activity at S1R included fluoxetine and fluvoxamine. SSRIs with intermediate affinity agonist activity at S1R included escitalopram and citalopram. SSRIs with low-affinity agonist activity at S1R included paroxetine.

^c^Antidepressants with FIASMA activity included amitriptyline, citalopram, clomipramine, desiparmine, doxepin, escitalopram, fluoxetine, fluvoxamine, imipramine, nortriptyline, paroxetine, sertraline, and venlafaxine.

^d^Antidepressants with unknown FIASMA activity included bupropion, desvenlafaxine, duloxetine, levomilnacipran, mirtazapine, nefazodone, trazodone, vilazodone, and vortioxetine.

Of the 25,034 patients, 2867 patients (11%) had an ED or hospital encounter within 30 days of the positive SARS-CoV-2 test result. In a crude analysis, the incidence of an ED or hospital encounter within 30 days was significantly greater among patients with antidepressant exposure than among patients without antidepressant exposure (971/4577 = 21.2%, versus 1896/20 457 = 9.3%, respectively, *p* < 0.001). However, after adjusting for all the potential confounding variables listed in Table 1, antidepressant exposure was associated with decreased odds of an ED or hospital encounter (adjusted odds ratio [AOR] 0.89, 95% CI 0.79–0.99, *p* = 0.04—Table 3), in a dose-dependent manner (in those taking <20 mg fluoxetine-equivalent daily: AOR 0.94, 95% CI 0.80–1.11, *p* = 0.48; in those taking ≥20 mg fluoxetine-equivalent daily: AOR 0.87, 95% CI 0.77–0.99, *p* = 0.04; in those taking ≥40 mg fluoxetine-equivalent daily: AOR 0.79, 95% CI 0.67–0.94, *p* = 0.006) (Table 4). Other independent predictors of an ED or hospital encounter in the logistic regression analysis included male sex, higher absolute value of the Elixhauser Index, greater number of home medications, non-White race or Hispanic ethnicity, and antipsychotic exposure (Table S3). The multivariable model had fair discrimination (c statistic = 0.78) and acceptable calibration (see calibration plot Fig. S1). All variance inflation factors were <2, demonstrating the absence of multicollinearity. In the sensitivity analysis removing 23 potentially influential outlier observations, antidepressant exposure remained associated with decreased odds of the primary outcome (Table S4). In the adjusted secondary analyses, SSRIs, antidepressants with FIASMA activity, and bupropion were associated with decreased odds of an ED or hospital encounter (Table 3). When patients were stratified according to their daily fluoxetine-equivalent doses, SSRIs and antidepressants with FIASMA activity were associated with decreased odds of the composite outcome only at daily fluoxetine-equivalent doses of at least 40 mg (Table 4). Bupropion was associated with decreased odds of the outcome at all doses (Table 4).

**Table 3:** Associations between exposure variables and ED or hospital encounter within 30 days.

Exposure	*N*^a^	Encounter		Unadjusted			Adjusted^b^		
		*N*	%	Odds ratio	95% CI	*p*	Odds ratio	95% CI	*p*
No Antidepressant	20,457	1896	9.3	Ref	Ref	Ref	Ref	Ref	Ref
Any Antidepressant	4577	971	21.2	2.64	2.42–2.87	<0.001	0.89	0.79–0.99	0.04
Any SSRI	2744	559	20.4	2.50	2.26–2.78	<0.001	0.87	0.75–0.99	0.04
Fluoxetine	403	84	20.8	2.58	2.02–3.29	<0.001	0.93	0.71–1.22	0.62
Fluvoxamine	17	4	23.5	3.01	0.98–9.25	0.05	0.79	0.24–2.54	0.69
Citalopram	507	103	20.3	2.50	2.00–3.11	<0.001	0.90	0.71–1.16	0.42
Escitalopram	812	157	19.3	2.35	1.96–2.81	<0.001	0.79	0.64–0.98	0.03
Paroxetine	179	34	19	2.30	1.58–3.34	<0.001	0.67	0.45–1.01	0.05
Sertraline	843	181	21.5	2.68	2.26–3.18	<0.001	0.92	0.75–1.12	0.41
Vilazodone	38	14	36.8	5.71	2.95–11.06	<0.001	1.63	0.80–3.34	0.18
Vortioxetine	52	12	23.1	2.94	1.54–5.61	0.001	0.85	0.43–1.72	0.66
Any Non-SSRI	2591	614	23.7	3.04	2.75–3.37	<0.001	0.93	0.82–1.06	0.28
Any TCA	567	152	26.8	3.59	2.96–4.34	<0.001	1.03	0.84–1.28	0.75
Amitriptyline	333	92	27.6	3.74	2.93–4.77	<0.001	1.10	0.85–1.44	0.47
Clomipramine	7	2	28.6	3.92	0.76–20.20	0.10	0.66	0.12–3.55	0.62
Desipramine	8	0	0	NA	NA	NA	NA	NA	NA
Doxepin	31	8	25.8	3.41	1.52–7.62	0.003	0.88	0.38–2.04	0.77
Imipramine	12	4	33.3	4.89	1.47–16.27	0.010	1.51	0.44–5.19	0.51
Nortriptyline	188	53	28.2	3.84	2.79–5.30	<0.001	1.13	0.81–1.59	0.47
Any Phenylpiperazine	761	203	26.7	3.56	3.01–4.21	<0.001	1.00	0.82–1.22	0.99
Nefazodone	2	0	0	NA	NA	NA	NA	NA	NA
Trazodone	760	203	26.7	3.57	3.02–4.22	<0.001	1.00	0.82–1.22	0.98
Any SNRI	994	226	22.7	2.88	2.47–3.37	<0.001	0.88	0.73–1.06	0.17
Desvenlafaxine	46	13	28.3	3.86	2.03–7.34	<0.001	1.36	0.69–2.71	0.38
Duloxetine	559	136	24.3	3.15	2.58–3.84	<0.001	0.89	0.71–1.11	0.30
Levomilnacipran	4	1	25	3.26	0.34–31.39	0.31	1.13	0.11–11.82	0.92
Venlafaxine	400	81	20.2	2.49	1.94–3.19	<0.001	0.81	0.61–1.07	0.13
Other Antidepressant									
Bupropion	589	103	17.5	2.07	1.67–2.58	<0.001	0.70	0.55–0.90	0.005
Mirtazapine	165	60	36.4	5.59	4.06–7.71	<0.001	1.22	0.86–1.74	0.27
SSRIs GROUPED BY S1R AFFINITY^c^									
High affinity	418	88	21.1	2.61	2.05–3.32	<0.001	0.93	0.72–1.22	0.61
Intermediate affinity	1307	257	19.7	2.40	2.07–2.77	<0.001	0.83	0.70–0.99	0.04
Low affinity	179	34	19	2.30	1.58–3.34	<0.001	0.67	0.45–1.01	0.05
ANTIDEPRESSANTS GROUPED BY FIASMA ACTIVITY									
With FIASMA activity^d^	3414	707	20.7	2.56	2.32–2.81	<0.001	0.87	0.77–0.99	0.03
With unknown FIASMA activity^e^	1904	461	24.2	3.13	2.79–3.51	<0.001	0.94	0.81–1.09	0.39

*CI* confidence interval, *FIASMA* functional inhibition of acid sphingomyelinase, *SNRI* serotonin-norepinephrine reuptake inhibitor, *SSRI* selective serotonin reuptake inhibitor, *S1R* sigma-1 receptor, *TCA* tricyclic antidepressant.

^a^*N* represent the number of patients included during calculation of the unadjusted odds ratio. In the multivariable logistic regression used to obtain the adjusted odds ratios, no imputation of missing values was performed and only complete cases were included. For example, in the primary analysis of exposure to any antidepressant versus exposure to no antidepressant, the total number of patients in the multivariable logistic regression was *N* = 21,051.

^b^Models were adjusted for sex, age, race and ethnicity, obesity, number of diagnoses, history of mood or anxiety disorder, history of other psychiatric disorders, number of home medications, concurrent exposure to benzodiazepine or Z drug, concurrent exposure to antipsychotic drugs, and concurrent exposure to non-antidepressant drugs with FIASMA or S1R activity (listed in Table S1).

^c^SSRIs with high-affinity agonist activity at S1R included fluoxetine and fluvoxamine. SSRIs with intermediate affinity agonist activity at S1R included escitalopram and citalopram. SSRIs with low-affinity agonist activity at S1R included paroxetine.

^d^Antidepressants with FIASMA activity included amitriptyline, citalopram, clomipramine, desiparmine, doxepin, escitalopram, fluoxetine, fluvoxamine, imipramine, nortriptyline, paroxetine, sertraline, and venlafaxine.

^e^Antidepressants with unknown FIASMA activity included bupropion, desvenlafaxine, duloxetine, levomilnacipran, mirtazapine, nefazodone, trazodone, vilazodone, and vortioxetine.

**Table 4:** Associations between selected antidepressant exposures and ED or hospital encounter within 30 days, stratified by fluoxetine-equivalent dose^a^.

Exposure	N	Encounters		Unadjusted			Adjusted^b^		
		n	%	Odds ratio	95% CI	*P*	Odds ratio	95% CI	*p*
Any Antidepressant									
No antidepressant	20,457	1896	9.3	Ref	Ref	Ref	Ref	Ref	Ref
<20 mg Fluoxetine-equivalent	1140	261	22.9	2.91	2.51–3.36	<0.001	0.94	0.80–1.11	0.48
≥20 mg Fluoxetine-equivalent	3437	710	20.7	2.55	2.32–2.80	<0.001	0.87	0.77–0.99	0.04
≥40 mg Fluoxetine-equivalent	1684	345	20.5	2.52	2.22–2.87	<0.001	0.79	0.67–0.94	0.006
Any SSRI									
No antidepressant	20,457	1896	9.3	Ref	Ref	Ref	Ref	Ref	Ref
<20 mg Fluoxetine-equivalent	321	62	19.3	2.34	1.77–3.10	<0.001	0.81	0.60–1.10	0.18
≥20 mg Fluoxetine-equivalent	2423	497	20.5	2.53	2.26–2.82	<0.001	0.88	0.77–1.02	0.09
≥40 mg Fluoxetine-equivalent	1049	210	20	2.45	2.09–2.87	<0.001	0.81	0.67–0.98	0.03
Bupropion^c^									
No antidepressant	20,457	1896	9.3	Ref	Ref	Ref	Ref	Ref	Ref
<20 mg Fluoxetine-equivalent	362	64	17.7	2.10	1.60–2.77	<0.001	0.71	0.52–0.95	0.02
≥20 mg Fluoxetine-equivalent	227	39	17.2	2.03	1.43–2.88	<0.001	0.68	0.47–0.99	0.04
Antidepressants with FIASMA Activity^d^									
No Antidepressant	20,457	1896	9.3	Ref	Ref	Ref	Ref	Ref	Ref
<20 mg Fluoxetine-equivalent	718	157	21.9	2.74	2.28–3.29	<0.001	0.89	0.73–1.09	0.25
≥20 mg Fluoxetine-equivalent	2696	550	20.4	2.51	2.26–2.79	<0.001	0.88	0.77–1.01	0.07
≥40 mg Fluoxetine-equivalent	1178	229	19.4	2.36	2.03–2.75	<0.001	0.78	0.65–0.94	0.009

*CI* confidence interval, *FIASMA* functional inhibition of acid sphingomyelinase, *SSRI* selective serotonin reuptake inhibitor,

^a^Analyses stratified by fluoxetine-equivalent dose were performed for classes of antidepressants and individual drugs (if not already included in one of the classes) that were significantly associated with reduced ED or hospital encounters (adjusted *p* value < 0.05 in Table 3).

^b^Models were adjusted for sex, age, race and ethnicity, obesity, number of diagnoses, history of mood or anxiety disorder, history of other psychiatric disorders, number of home medications, concurrent exposure to benzodiazepine or Z drug, concurrent exposure to antipsychotic drugs, and concurrent exposure to non-antidepressant drugs with FIASMA or S1R activity (listed in Table S1).

^c^Bupropion not tested at ≥40 mg fluoxetine-equivalent due to small number of cases (*N* = 5).

^d^Antidepressants with FIASMA activity included amitriptyline, citalopram, clomipramine, desiparmine, doxepin, escitalopram, fluoxetine, fluvoxamine, imipramine, nortriptyline, paroxetine, sertraline, and venlafaxine.

## Discussion

In this single-center, retrospective cohort of 25,034 ambulatory patients infected with SARS-CoV-2, exposure to an antidepressant was significantly associated with reduced incidence of ED visitation or hospital admission after adjusting for sociodemographic characteristics, medical comorbidity, psychiatric conditions, and concurrent treatments, with a significant dose-dependent relationship. In secondary analyses examining specific classes of antidepressants, the benefit was observed with exposure to SSRIs, antidepressants with FIASMA activity, and bupropion. This association was only observed among patients exposed to a dose of antidepressants of at least 20 mg of fluoxetine-equivalents per day and was stronger for doses of at least 40 mg of fluoxetine-equivalents per day.

The results of this study are broadly consistent with recently published literature examining clinical deterioration in patients with COVID-19 exposed to pre-illness SSRIs. In a multicenter, retrospective observational study of 7230 adult patients who were hospitalized for COVID-19 in Paris, antidepressant use, particularly fluoxetine, paroxetine, escitalopram, venlafaxine, and mirtazapine, was associated with reduced risk of intubation or death [19]. In a subsequent multicenter, retrospective cohort study of 83,584 patients with COVID-19 treated in hospitals, EDs, or urgent care clinics in the United States, pre-illness SSRI use was associated with a reduced relative risk of mortality, with the largest effects observed in patients taking fluoxetine [20]. This study similarly demonstrates that pre-illness use of antidepressants as a whole, and SSRIs in particular, is associated with reduced risk of ED visitations or hospital admission in ambulatory patients infected with SARS-CoV-2. On the other hand, some studies have reported conflicting results. Notably, a meta-analysis found no significant association between antidepressant use and mortality [37]. However, a critical limitation for interpreting this finding is that estimates included were adjusted for age, sex and only for a limited number of comorbid medical conditions, and did not take into account clinical severity, co-prescriptions, obesity, and medical indications for antidepressants, so residual confounding may be at work and possibly mask the association [32].

The largest effect was seen with pre-illness exposure to the highest dose of SSRIs, hereby stratified as a daily dose ≥40 mg of fluoxetine-equivalents. This finding could be in line with results of a prior meta-analysis showing that most antidepressants, mainly SSRIs, are significantly associated with reduced levels of several pro-inflammatory cytokines (i.e. IL-6, IL-10, TNF-alpha, CCL-2) among depressed individuals [38]. Given that elevations in these cytokines are associated with severe COVID-19, any protective effect of SSRIs may plausibly act through immunomodulation.

Examination of additional subgroups in this study permitted inferences on the mechanisms by which SSRIs and other antidepressants may provide a protective effect during SARS-CoV-2 infection. The incidence of ED visitation or hospital admission was significantly reduced among patients exposed to antidepressants with FIASMA activity (including paroxetine). Acid sphingomyelinase (ASM) plays a key role in the transduction of pathological signals into the cell. Hydrolysis of sphingomyelin, catalyzed by ASM, results in the production of ceramide, a lipophilic sphingolipid that gathers within the outer layer of the cell membrane. “Rafts” of ceramide allow necessary receptor proteins to aggregate, facilitating pathological signal transduction [27, 39]. Antidepressant-mediated inhibition of ASM may provide protection against COVID-19 both via reduced viral entry into cells (due to reduced formation of ceramide-enriched membrane domains) and via reduced inflammatory response [6, 16]. The use of antidepressants with FIASMA activity against SARS-CoV-2 infection has been studied in preclinical models [10] and in a multicenter retrospective cohort study. Among individuals hospitalized with severe COVID-19, the use of a medication with FIASMA activity was associated with reduced hazards of intubation or death [35]. Similar results were observed in another cohort of individuals with psychiatric disorders hospitalized for severe COVID-19 [21]. In a retrospective cohort study conducted at an adult psychiatric facility, exposure to antidepressants was associated with a reduced incidence of COVID-19 infection [40], and a similar result was found for fluoxetine in a large pharmacopeia-wide association study [41]. Additionally, ceramide levels, sphingomyelinase activity, and ceramidase activity have all been correlated with COVID-19 severity and with inflammatory markers [42–45], lending further support for FIASMA as an explanatory mechanism behind the associations reported in observational studies.

In contrast, no dose-response effect was observed in this cohort when the SSRIs were stratified according to their level of S1R affinity. The relatively high adjusted odds ratio for the one S1R *antagonist*, sertraline, suggests it may be less protective than other SSRIs; however, the confidence intervals for all SSRI odds ratios overlap, and therefore, the strengths of the effects cannot be distinguished. Although the S1R-mediated anti-inflammatory effects of fluvoxamine have been studied in some detail, the effects of other SSRIs on the S1R are not as well understood. Additionally, the beneficial effects of SSRIs and non-SSRI agents with serotonin antagonism may be time-dependent in COVID-19, owing to their diverse anti-platelet [46] and serotonin modulating properties [47]. On one hand, early or pre-illness use of SSRIs may be beneficial by diminishing the degree of serotonin loading onto mature platelets during the viral replication phase [48], before the onset of severe platelet activation and pathogenic platelet mediator release that are hallmarks of the inflammatory phase in COVID-19. Thus, early or pre-illness SSRI use preemptively reduces a key content of platelet granules, serotonin, which is demonstrated to cause pathogenicity in illnesses driven by immune-mediated platelet activation [49] and also drives immunothrombosis in severe COVID-19 [50]. On the other hand, the direct serotonin receptor inhibitory effects of some SSRIs and non-SSRI agents (e.g., bupropion) may be beneficial even after the inflammatory phase has begun [40, 49, 51], by inhibiting the pro-thrombotic and pro-inflammatory action of released serotonin from severely activated platelets in this illness. Further research is necessary to explore the optimal timing of treatment with serotonin modulating agents. If antidepressants are demonstrated to have multiple modalities of action as discussed above, their benefit may not be limited to a specific phase of illness, unlike antivirals (best if given in the first 5 days of symptoms) and systemic steroids (if given too early, could suppress the appropriate immune response to the virus) [52, 53].

Interestingly, exposure to bupropion, a non-SSRI dopamine, and norepinephrine reuptake inhibitor, was significantly associated with improved outcomes in this study. Although we cannot rule out the possibility that this association may result from multiple testing or that the pattern of confounding may be different for bupropion (often used for indications other than depression compared to other antidepressants), its effect on ASM has never been formally tested, to our knowledge. However, bupropion may have S1R agonist activity, although this has been incompletely evaluated [54]. It is also a negative allosteric modulator of serotonin type 3 A (5HT3A) receptors [55]. Another 5HT3 antagonist, ondansetron, was associated with better outcomes, including reduced mortality in a study of COVID-19 inpatients [51]. Therefore, it may be worthwhile to further explore the repurposing of 5HT3A antagonists as treatment for COVID-19. Blocking the 5HT3A receptor with the selective antagonist MDL 72222 totally prevented adverse respiratory effects of exogenous serotonin administration in a study of cats [56]. Relevant to this, a majority of patients in the inflammatory phase of COVID-19 experience severe platelet activation and excessive platelet aggregates [57], and consequently platelet serotonin is acutely released from platelet granules into plasma. Since plasma serotonin in patients with COVID-19 reaches levels several folds beyond normal, and even significantly beyond levels seen in other etiologies of acute respiratory distress syndrome [58], early blockage of the 5HT3A receptor may minimize adverse effects of excessive platelet serotonin release, perhaps preventing or ameliorating respiratory deterioration and other potential complications of sustained plasma serotonin rise in COVID-19. Lastly, other mechanisms such as bupropion’s ability to lower the levels of the inflammatory mediators TNF-alpha and interferon-gamma in preclinical models may additionally contribute to the beneficial association demonstrated in this study [59].

Of note, the incidence of antidepressant exposure in this cohort (18.3%) was higher than the incidence reported in previous cohorts. Between 2015 and 2018, the National Health and Nutrition Examination Survey found that 13.2% of American adults used an antidepressant within the past 30 days [60]. The higher incidence reported in this manuscript may reflect the well-documented increase in both depressive symptoms [61] and antidepressant use [62] since the start of the pandemic. Another possibility is that individuals who take antidepressants may have been more likely to develop COVID-19 compared to other community-dwelling adults, resulting in selection bias. Alternatively, individuals taking antidepressants may have been more likely to have their SARS-CoV-2 testing performed within the university-affiliated healthcare system rather than at other testing sites in the community.

One of the strengths of this work is the rigorous adjustment for confounding variables employed in the analysis. When compared to patients without antidepressant exposure, patients with antidepressant exposure were older, had more diagnoses including other psychiatric diagnoses, and took more medications at home including other psychoactive medications (Table 1). Thus, antidepressant exposure would act as a surrogate marker for overall degree of comorbidity if proper risk adjustments were not performed. This explains why antidepressant exposure was associated with increased risk of ED visitation or hospital admission in the initial unadjusted analysis. A comprehensive risk adjustment, however, not only eliminated this ostensible increased risk, but instead revealed an association in the opposite direction toward protection against ED visitation or hospital admission in those with antidepressant exposure.

This study also has limitations that should be noted. First, this was an observational study, and therefore the observed associations should not be interpreted as causal effects. Residual confounding cannot be ruled out, especially because patients with depression may have different patterns of healthcare utilization compared to other patients with similar degree of physical illness [63]. However, the presence of biologically plausible mechanisms for the observed associations and the agreement with other recent studies reinforces the validity of our findings and posits that prospective interventional studies of SSRIs other than fluvoxamine may be appropriate for the early treatment of COVID-19. Second, information on vital status was unavailable unless patients died while in the hospital, which prevented the addition of death to the composite outcome. Third, medication daily dosages were approximated using the formulation strength because prescription sig data were not available. Although this is not a perfect approximation, it is likely reasonable because the medications included in the analysis are usually prescribed as one tablet/capsule once daily. Fourth, medication start and stop dates were not available, so the analyses could not adjust for the duration of antidepressant use. Fifth, this analysis was conducted at a single healthcare system. Although the healthcare system in this study has a wide footprint, including academic and non-academic hospitals that serve both urban and rural communities, these findings may not be generalizable to other settings. However, the congruence of our observations with the findings of other recent studies mitigate the concerns about generalizability. Sixth, some patients may have sought ED or inpatient care at facilities outside this healthcare system, and these visits would not have been detected. Seventh, information about vaccination was not available. Finally, the magnitude of the associations may be underestimated given the high rate of antidepressant discontinuation in the clinical outpatient setting [64].

In conclusion, pre-illness exposure to antidepressants was associated with a decreased odds of ED visitation or hospital admission among ambulatory patients infected with SARS-CoV-2. This association appears to be related to exposure to SSRIs and in particular to the agents with FIASMA activity. When designing future prospective studies, researchers should consider SSRIs and other antidepressants with FIASMA activity as candidate interventions for outpatient therapy of COVID-19, prescribed at a dose of at least 40 mg/day of fluoxetine-equivalents.

## Supplementary information

Supplementary Online Content
