# Supplementary material for: Association between antidepressant use and ED or hospital visits in outpatients with SARS-CoV-2
Source: Transl Psychiatry. 2022 Aug 22;12:341. doi: 10.1038/s41398-022-02109-3 (PMC9395392; doi:10.1038/s41398-022-02109-3)
Supplement: Supplementary file 1 — Supplementary Online Content [file 41398_2022_2109_MOESM1_ESM.docx]

**Supplementary Online Content**

**“Association Between Antidepressant Use and ED or Hospital Visits in Outpatients with SARS-CoV-2”**

Table S1. Medications Retrieved from Home Medication List

Table S2. Conversion Factors Used to Calculate Fluoxetine-Equivalent Dose

Table S3. Logistic Regression for ED or Hospital Encounter within 30 Days

Figure S1. Calibration Plot for Logistic Regression

Table S4. Sensitivity Analysis - Logistic Regression for ED or Hospital Encounter within 30 Days after Removing Influential Outliers

**Table S1. Medications Retrieved from Home Medication List**

| **Medication Class** | **Medications Included** |
| --- | --- |
| Selective Serotonin Reuptake Inhibitors | Citalopram, Escitalopram, Fluoxetine, Fluvoxamine, Paroxetine, Sertraline |
| Tricyclic Antidepressants | Amitriptyline, Clomipramine, Desipramine, Doxepin, Imipramine, Nortriptyline |
| Phenylpiperazines | Nefazodone, Trazodone |
| Serotonin Norepinephrine Reuptake Inhibitors | Desvenlafaxine, Duloxetine, Levomilnacipran, Venlafaxine |
| Other Antidepressants | Bupropion, Mirtazapine |
| Benzodiazepines | Alprazolam, Chlordiazepoxide, Clorazepate, Diazepam, Flurazepam, Lorazepam, Oxazepam, Temazepam, Triazolam |
| Z Drugs | Eszopiclone, Zaleplon, Zolpidem |
| Antipsychotics | Aripiprazole, Asenapine, Brexpiprazole, Cariprazine, Chlorpromazine, Clozapine, Droperidol, Fluphenazine, Haloperidol, Iloperidone, Loxapine, Lurasidone, Olanzapine, Paliperidone, Perphenazine, Pimavanserin, Pimozide, Quetiapine, Risperidone, Thiothiene, Ziprasidone |
| Non-Antidepressant Drugs with Functional Inhibition of Acid Sphingomyelinase Activity | Amiodarone, Amlodipine, Aripiprazole, Benztropine, Carvedilol, Chlorpromazine, Clemastine, Clomipramine, Cyclobenzaprine, Cyproheptadine, Desloratadine, Hydroxyzine, Loperamide, Loratadine, Perphenazine, Pimozide, Promethazine, Tamoxifen |
| Non-Antidepressant Drugs with Sigma-1 Receptor Activity | Chlorpromazine, Donepezil, Fluphenazine, Haloperidol, Perphenazine, Pimozide |

**Table S2. Conversion Factors Used to Calculate Fluoxetine-Equivalent Dose**

| **Antidepressant** | **Conversion Factor** |
| --- | --- |
| Fluoxetine | 1 |
| Fluvoxamine | 0.3 |
| Citalopram | 1.11 |
| Escitalopram | 2.22 |
| Paroxetine | 1.17 |
| Sertraline | 0.42 |
| Vilazodone | 1.5 |
| Vortioxetine | 3 |
| Amitriptyline | 0.33 |
| Clomipramine | 0.35 |
| Desipramine | 0.21 |
| Doxepin | 0.29 |
| Imipramine | 0.29 |
| Nortriptyline | 0.40 |
| Nefazodone | 0.08 |
| Trazodone | 0.10 |
| Desvenlafaxine | 0.40 |
| Duloxetine | 0.67 |
| Levomilnacipran | 0.33 |
| Venlafaxine | 0.28 |
| Bupropion | 0.11 |
| Mirtazapine | 0.79 |

**Table S3. Logistic Regression for ED or Hospital Encounter within 30 Days**

| **Variable** | **N ^a^** | **Encounter within 30 Days** | | **Unadjusted** | | | **Adjusted** | | | **VIF** |
| --- | --- | --- | --- | --- | --- | --- | --- | --- | --- | --- |
|  |  | **N** | **%** | **Odds Ratio** | **95% CI** | **p** | **Odds Ratio** | **95% CI** | **p** |  |
| Any Antidepressant |  |  |  |  |  |  |  |  |  | 1.59 |
| FALSE | 20 457 | 1 896 | 9.3 | Ref | Ref |  | Ref | Ref |  |  |
| TRUE | 4 577 | 971 | 21.2 | 2.64 | 2.42-2.87 | < 0.001 | 0.89 | 0.79-0.99 | 0.04 |  |
| Sex |  |  |  |  |  |  |  |  |  | 1.08 |
| Female | 15 030 | 1 676 | 11.2 | Ref | Ref |  | Ref | Ref |  |  |
| Male | 9 986 | 1 191 | 11.9 | 1.08 | 1.00-1.17 | 0.06 | 1.32 | 1.21-1.44 | < 0.001 |  |
| Number of Medications |  |  |  |  |  |  |  |  |  | 1.80 |
| 0 | 11 149 | 126 | 1.1 | Ref | Ref |  | Ref | Ref |  |  |
| 01-05 | 4 736 | 553 | 11.7 | 11.57 | 9.50-14.08 | < 0.001 | 8.83 | 7.18-10.85 | < 0.001 |  |
| 06-10 | 3 435 | 568 | 16.5 | 17.33 | 14.23-21.11 | < 0.001 | 12.28 | 9.93-15.19 | < 0.001 |  |
| 11-15 | 2 382 | 530 | 22.3 | 25.04 | 20.49-30.59 | < 0.001 | 17.09 | 13.68-21.35 | < 0.001 |  |
| 16+ | 3 332 | 1 090 | 32.7 | 42.53 | 35.17-51.43 | < 0.001 | 26.47 | 21.10-33.20 | < 0.001 |  |
| Obese |  |  |  |  |  |  |  |  |  | 1.04 |
| FALSE | 10 603 | 1 242 | 11.7 | Ref | Ref |  | Ref | Ref |  |  |
| TRUE | 11 197 | 1 619 | 14.5 | 1.27 | 1.18-1.38 | < 0.001 | 0.96 | 0.88-1.05 | 0.34 |  |
| White Non-Hispanic |  |  |  |  |  |  |  |  |  | 1.12 |
| FALSE | 8 524 | 1 173 | 13.8 | Ref | Ref |  | Ref | Ref |  |  |
| TRUE | 15 507 | 1 686 | 10.9 | 0.76 | 0.71-0.83 | < 0.001 | 0.58 | 0.53-0.63 | < 0.001 |  |
| Mood or Anxiety Disorder |  |  |  |  |  |  |  |  |  | 1.54 |
| FALSE | 17 296 | 1 848 | 10.7 | Ref | Ref |  | Ref | Ref |  |  |
| TRUE | 5 884 | 1 014 | 17.2 | 1.74 | 1.60-1.89 | < 0.001 | 0.98 | 0.88-1.10 | 0.77 |  |
| Other Psychiatric Disorder |  |  |  |  |  |  |  |  |  | 1.11 |
| FALSE | 20 763 | 2 417 | 11.6 | Ref | Ref |  | Ref | Ref |  |  |
| TRUE | 2 417 | 445 | 18.4 | 1.71 | 1.53-1.91 | < 0.001 | 0.89 | 0.79-1.01 | 0.07 |  |
| Benzodiazepine or Z Drug |  |  |  |  |  |  |  |  |  | 1.16 |
| FALSE | 23 037 | 2 369 | 10.3 | Ref | Ref |  | Ref | Ref |  |  |
| TRUE | 1 997 | 498 | 24.9 | 2.90 | 2.60-3.24 | < 0.001 | 1.13 | 1.00-1.27 | 0.06 |  |
| Antipsychotic Drug |  |  |  |  |  |  |  |  |  | 1.13 |
| FALSE | 24 452 | 2 671 | 10.9 | Ref | Ref |  | Ref | Ref |  |  |
| TRUE | 582 | 196 | 33.7 | 4.14 | 3.47-4.94 | < 0.001 | 1.50 | 1.24-1.83 | < 0.001 |  |
| Non-Antidepressant Drug with FIASMA or S1R Activity |  |  |  |  |  |  |  |  |  | 1.27 |
| FALSE | 19 989 | 1 511 | 7.6 | Ref | Ref |  | Ref | Ref |  |  |
| TRUE | 5 045 | 1 356 | 26.9 | 4.50 | 4.14-4.88 | < 0.001 | 1.24 | 1.13-1.37 | < 0.001 |  |
| Age (per year) | 25 034 |  |  | 1.02 | 1.02-1.02 | < 0.001 | 1.00 | 1.00-1.00 | 0.45 | 1.37 |
| Absolute Value of Elixhauser Index (per unit) | 23 180 |  |  | 1.12 | 1.11-1.13 | < 0.001 | 1.03 | 1.02-1.04 | < 0.001 | 1.20 |

Abbreviation: FIASMA = functional inhibition of acid sphingomyelinase. S1R = sigma-1 receptor. VIF = variance inflation factor

^a^ N represent the number of patients included during calculation of the unadjusted odds ratio. In the multivariable logistic regression used to obtain the adjusted odds ratios, no imputation of missing values was performed and only complete cases were included. The total number of patients in the multivariable logistic regression was N = 21,051.

**Figure S1. Calibration Plot for Multivariable Logistic Regression**


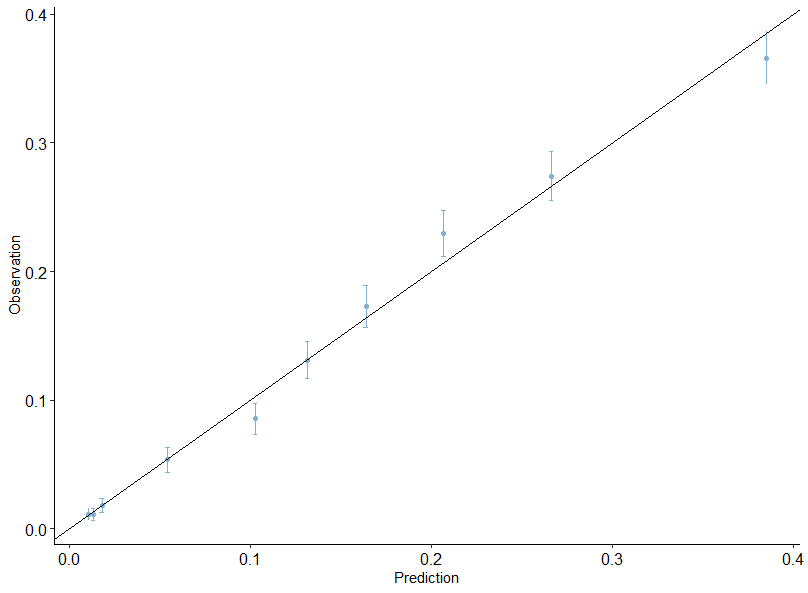


This figure demonstrates the calibration of the logistic regression model shown in Table S3.

**Table S4. Sensitivity Analysis - Logistic Regression for ED or Hospital Encounter within 30 Days after Removing Influential Outliers ^a^**

| **Variable** | **N ^b^** | **Encounter within 30 Days** | | **Unadjusted** | | | **Adjusted** | | |
| --- | --- | --- | --- | --- | --- | --- | --- | --- | --- |
|  |  | **N** | **%** | **Odds Ratio** | **95% CI** | **p** | **Odds Ratio** | **95% CI** | **p** |
| Any Antidepressant |  |  |  |  |  |  |  |  |  |
| FALSE | 20 434 | 1 873 | 9.2 | Ref | Ref |  | Ref | Ref |  |
| TRUE | 4 577 | 971 | 21.2 | 2.67 | 2.45-2.91 | < 0.001 | 0.89 | 0.80-1.00 | 0.047 |
| Sex |  |  |  |  |  |  |  |  |  |
| Female | 15 007 | 1 653 | 11 | Ref | Ref |  | Ref | Ref |  |
| Male | 9 986 | 1 191 | 11.9 | 1.09 | 1.01-1.18 | 0.03 | 1.36 | 1.24-1.48 | < 0.001 |
| Number of Medications |  |  |  |  |  |  |  |  |  |
| 0 | 11 126 | 103 | 0.9 | Ref | Ref |  | Ref | Ref |  |
| 01-05 | 4 736 | 553 | 11.7 | 14.15 | 11.43-17.51 | < 0.001 | 11.10 | 8.86-13.92 | < 0.001 |
| 06-10 | 3 435 | 568 | 16.5 | 21.20 | 17.12-26.26 | < 0.001 | 15.51 | 12.30-19.56 | < 0.001 |
| 11-15 | 2 382 | 530 | 22.3 | 30.63 | 24.66-38.04 | < 0.001 | 21.66 | 17.02-27.56 | < 0.001 |
| 16+ | 3 332 | 1 090 | 32.7 | 52.03 | 42.30-64.00 | < 0.001 | 33.60 | 26.30-42.91 | < 0.001 |
| Obese |  |  |  |  |  |  |  |  |  |
| FALSE | 10 594 | 1 233 | 11.6 | Ref | Ref |  | Ref | Ref |  |
| TRUE | 11 183 | 1 605 | 14.4 | 1.27 | 1.17-1.38 | < 0.001 | 0.95 | 0.87-1.04 | 0.25 |
| White Non-Hispanic |  |  |  |  |  |  |  |  |  |
| FALSE | 8 524 | 1 173 | 13.8 | Ref | Ref |  | Ref | Ref |  |
| TRUE | 15 484 | 1 663 | 10.7 | 0.75 | 0.70-0.82 | < 0.001 | 0.56 | 0.51-0.62 | < 0.001 |
| Mood or Anxiety Disorder |  |  |  |  |  |  |  |  |  |
| FALSE | 17 276 | 1 828 | 10.6 | Ref | Ref |  | Ref | Ref |  |
| TRUE | 5 881 | 1 011 | 17.2 | 1.75 | 1.61-1.91 | < 0.001 | 0.99 | 0.89-1.10 | 0.83 |
| Other Psychiatric Disorder |  |  |  |  |  |  |  |  |  |
| FALSE | 20 743 | 2 397 | 11.6 | Ref | Ref |  | Ref | Ref |  |
| TRUE | 2 414 | 442 | 18.3 | 1.72 | 1.53-1.92 | < 0.001 | 0.88 | 0.78-1.00 | 0.05 |
| Benzodiazepine or Z Drug |  |  |  |  |  |  |  |  |  |
| FALSE | 23 014 | 2 346 | 10.2 | Ref | Ref |  | Ref | Ref |  |
| TRUE | 1 997 | 498 | 24.9 | 2.93 | 2.62-3.27 | < 0.001 | 1.13 | 1.00-1.28 | 0.05 |
| Antipsychotic Drug |  |  |  |  |  |  |  |  |  |
| FALSE | 24 429 | 2 648 | 10.8 | Ref | Ref |  | Ref | Ref |  |
| TRUE | 582 | 196 | 33.7 | 4.18 | 3.50-4.98 | < 0.001 | 1.50 | 1.24-1.82 | < 0.001 |
| Non-Antidepressant Drug with FIASMA or S1R Activity |  |  |  |  |  |  |  |  |  |
| FALSE | 19 966 | 1 488 | 7.5 | Ref | Ref |  | Ref | Ref |  |
| TRUE | 5 045 | 1 356 | 26.9 | 4.56 | 4.21-4.95 | < 0.001 | 1.24 | 1.13-1.36 | < 0.001 |
| Age (per year) | 25 011 |  |  | 1.02 | 1.02-1.02 | < 0.001 | 1.00 | 1.00-1.00 | 0.27 |
| Absolute Value of Elixhauser Index (per unit) | 23 157 |  |  | 1.12 | 1.11-1.13 | < 0.001 | 1.03 | 1.02-1.04 | < 0.001 |

Abbreviation: FIASMA = functional inhibition of acid sphingomyelinase. S1R = sigma-1 receptor. VIF = variance inflation factor

^a^ Influential outliers were defined as observations with standardized residual > 3 and Cook’s distance > 4/n, where n is the total number of observations. A total of 23 influential outliers were identified in the dataset and removed for this sensitivity analysis.

^b^ N represent the number of patients included during calculation of the unadjusted odds ratio. In the multivariable logistic regression used to obtain the adjusted odds ratios, no imputation of missing values was performed and only complete cases were included. The total number of patients in the multivariable logistic regression was N = 21,028.
